# Management of a First Non-febrile Convulsive Seizure in the Pediatric Emergency Department: A Retrospective Study From 2015 to 2021 at the University Hospital of Reims

**DOI:** 10.7759/cureus.106050

**Published:** 2026-03-29

**Authors:** Amine Kaake, Mélanie Jennesson-Lyver

**Affiliations:** 1 Pediatric Neurology, Hospital Robert Debré Ap-Hp, Paris, FRA; 2 Pediatric Neurology, University Hospital of Reims, American Memorial Hospital, Reims, FRA

**Keywords:** childhood epilepsy, epilepsy in children, epileptic seizure, first seizure, pediatric emergency department, pediatric seizure

## Abstract

Introduction: The onset of epileptic manifestations frequently occurs during childhood and often leads to initial management in pediatric emergency departments. The diagnostic approach is challenging, as epileptic seizures must be distinguished from non-epileptic paroxysmal events and acute symptomatic seizures. Although several national and international recommendations exist, real-world data on the management of first seizures in pediatric emergency settings remain limited.

Objective: The primary objective of this study was to analyze patient characteristics and management of children presenting to a pediatric emergency department with a first non-febrile convulsive seizure. The secondary objective was to develop a practical management algorithm tailored to pediatric emergency settings.

Methods: We conducted a retrospective, single-center observational study in a tertiary pediatric hospital in Reims, France. All patients under 18 years of age presenting to the pediatric emergency department with a first non-febrile convulsive seizure between January 1, 2015, and June 30, 2021, were included.

Results: Data from 167 children were analyzed and categorized into three groups: epileptic seizure (99, 59.3%), non-epileptic paroxysmal event (64, 38.3%), and acute symptomatic seizure (4, 2.4%). Clinical examination was normal in the majority of cases and did not reliably discriminate between groups. Semiological features such as eye deviation, eye rolling, generalized or focal hypertonia, and postictal confusion were significantly associated with epileptic seizures, whereas stressful or vasovagal situations were more frequent in non-epileptic events.

All patients in the epileptic group underwent neuropediatric consultation, and 55 (55.5%) were discharged with antiepileptic treatment. Laboratory investigations were performed in 52 (52.5%) patients, with abnormalities identified in only 4% of cases. Electroencephalography (EEG) was performed in 96 (96.7%) patients and showed abnormalities in 64 (69.8%). In non-epileptic events, the EEG was normal in all cases where it was performed. Brain imaging was selectively performed and identified structural abnormalities in 21 (26.6%) patients who underwent MRI.

Conclusions: In children presenting with a first non-febrile convulsive event, epileptic seizures accounted for a substantial proportion of cases, while non-epileptic events remained frequent. Clinical history and witness description were the most informative elements for diagnosis, whereas routine laboratory testing had limited utility. EEG and neuroimaging were valuable in selected cases.

The proposed management algorithm provides a pragmatic, emergency-oriented framework to support clinical decision-making and help standardize the evaluation of these patients.

## Introduction

Epilepsy, one of the most common neurological disorders, affects approximately 50 million people worldwide, according to the World Health Organization [1]. It is not a single disease but a group of disorders characterized by excessive and abnormal synchronous neuronal activity in the brain, resulting in diverse epileptic seizures. Seizures can affect sensory, motor, autonomic functions, consciousness, emotional state, memory, cognition, or behavior [2].

The first manifestations of epilepsy often appear during childhood, making pediatric emergency departments the primary setting for initial seizure management. French guidelines were established in 2008 for managing first epileptic seizures in children. Since then, numerous recommendations have been published, including those issued by the French National Authority for Health (HAS) in 2020 for the management of epilepsy in children and adults [3], as well as the International League Against Epilepsy (ILAE) classification of epilepsies updated in 2017 [4,5].

The primary challenge in pediatric emergency management is differentiating epileptic seizures from other non-epileptic paroxysmal events such as syncope, breath-holding spells, and psychogenic non-epileptic seizures, as well as occasional seizures secondary to temporary causes such as fever, infection, electrolyte imbalance (e.g., dehydration), head trauma, or intoxication [4-6].

Accurate diagnosis requires a detailed history, including pregnancy and perinatal history, previous seizures, family history, and the child’s medical background. A comprehensive clinical and neurological examination is essential to identify treatable causes such as fever or altered consciousness.

Based on clinical findings, appropriate complementary investigations, including laboratory tests, imaging studies, and electroencephalography (EEG), are selected. If an epileptic seizure is confirmed, an individualized antiepileptic treatment plan is developed, taking into account the risk of recurrence and potential treatment-related adverse effects.

However, most existing studies and guidelines address first seizures in general and do not specifically focus on convulsive presentations in pediatric emergency settings, nor on real-world management practices. The aim of this study was to describe the management of children presenting with a first non-febrile convulsive event in a pediatric emergency department and to propose a pragmatic management algorithm based on both current recommendations and observed clinical practice.

## Materials and methods

Study design

This retrospective, single-center, observational study was conducted at the University Hospital of Reims in the Grand-Est region of France. Data were retrospectively collected from patients’ electronic medical records.

Study population and data

Patients were identified using ICD-10 diagnostic codes related to seizures and epilepsy, including G40 (epilepsy), G41 (status epilepticus), and R56.8 (other and unspecified convulsions). In order to reduce misclassification and capture potential differential diagnoses, additional ICD-10 codes corresponding to transient loss of consciousness and non-epileptic paroxysmal events were also screened, including R55 (syncope and collapse), R40.4 (transient alteration of awareness), and R25.8 (abnormal involuntary movements). Additional cases were identified through keyword searches ("convulsion," “seizure," "epilepsy," "alteration of awareness") in medical records. 

Patients were eligible for inclusion if they were under 18 years of age and presented with a first non-febrile convulsive seizure to the pediatric emergency department or were subsequently hospitalized in the Pediatric Short Stay Unit or Pediatric Medicine Departments at the American Memorial Hospital, University Hospital of Reims, between January 1, 2015, and June 30, 2021.

Exclusion criteria included patients younger than one month or older than 18 years, febrile seizures, and patients with a known history of epilepsy or already under neurological follow-up for epilepsy.

This case identification strategy may have preferentially captured convulsive presentations and may not fully reflect non-convulsive epileptic seizures.

Data extraction

Data were provided by the Medical Information Department using ICD-10 codes recorded during patient visits within the study period. In the absence of specific coding, keyword searches for “convulsion” were used to identify additional cases.

A total of 2037 patients were initially identified. Of these, 1870 (91.8%) were excluded: 1312 (64.4%) for febrile seizures, 538 (26.4%) for ongoing epilepsy, and 20 (1.0%) due to significant missing data. The final study population included 167 (8.2%) patients.

Data collection

Data were retrospectively extracted from medical records by an investigator using a standardized data collection form. For each patient, we collected demographic characteristics, medical history, circumstances of seizure onset, clinical examination findings, suspected seizure type, diagnostic strategy implemented in the emergency department or during hospitalization, and complementary investigations (laboratory, imaging, and electrophysiological tests). Final diagnosis and treatment at discharge were also recorded.

Data were obtained through a comprehensive review of electronic medical records (Easily®, Hopsis, Aix-en-Provence, France, and Urqual®, Mipih, Paris, France) and compiled into Microsoft Excel (Microsoft Corporation, Redmond, WA). Missing data were not imputed, and analyses were performed on available cases. Given the retrospective design, the study is subject to potential information bias and misclassification.

Study population

**Figure 1:**
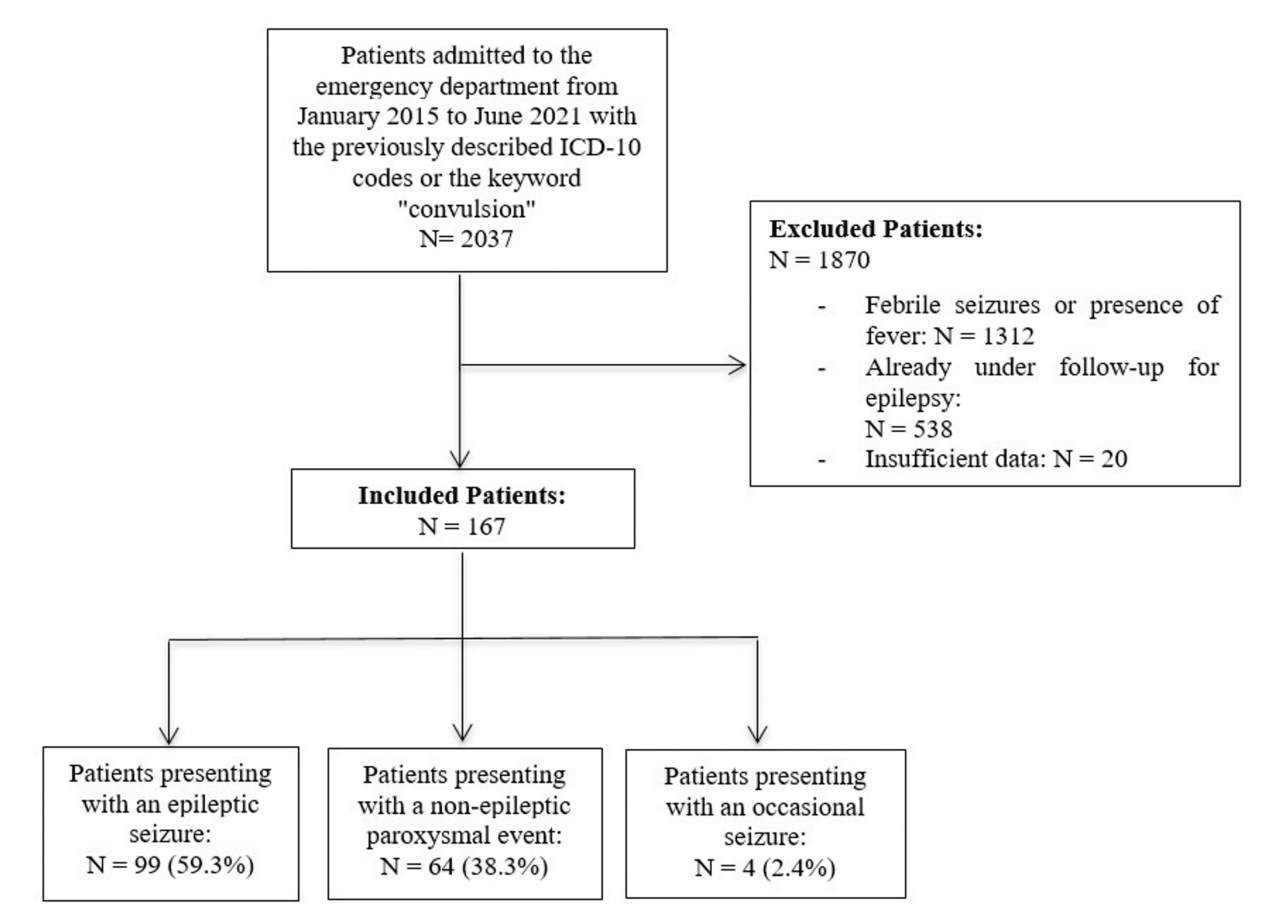
Flowchart of the included study population The flowchart (Figure 1) outlines the study population divided into three groups. The first group included 99 (59.3%) patients who presented with an epileptic seizure, the second group comprised 64 (38.3%) patients who experienced a non-epileptic paroxysmal event, and the third group included 4 (2.4%) patients with acute symptomatic seizures.

The study selection process is summarized in Figure 1. The final study population was divided into three diagnostic groups: epileptic seizures (n=99, 59.3%), non-epileptic paroxysmal events (n=64, 38.3%), and acute symptomatic seizures (n=4, 2.4%).

Statistical analysis

Statistical analysis was performed using Microsoft Excel, version 2024 (Microsoft Corp., Redmond, WA). Categorical variables were expressed as numbers and percentages (n, %). The analysis was primarily descriptive, with comparisons between groups based on crude associations. No multivariable analysis was performed. Comparisons between the three diagnostic groups were conducted using Fisher’s exact test (Freeman-Halton extension) due to small expected cell counts. For selected variables, crude odds ratios (OR) with 95% confidence intervals (CI) were calculated to assess associations between clinical features and epileptic seizures. For these analyses, patients were dichotomized into epileptic seizures versus non-epileptic and acute symptomatic seizures combined. When zero cell counts were present, the Haldane-Anscombe correction was applied. All tests were two-sided, and a p-value < 0.05 was considered statistically significant. Given the sample size and study design, results should be interpreted as exploratory.

Regulatory framework

This study was conducted in accordance with the General Data Protection Regulation (GDPR) and was declared to and approved by the Data Protection Officer of the University Hospital of Reims under protocol MR004. The authors declare no funding and no conflicts of interest.

Management algorithm

The management algorithm was developed by the authors based on current international recommendations and the results of this study, with the aim of providing a pragmatic, emergency-oriented framework for clinical practice. The algorithm was created using Microsoft PowerPoint.

## Results

Patient diagnosis

A diagnosis of epileptic syndrome according to the 2017 ILAE classification was established in 99 (59.3%) patients: idiopathic generalized epilepsies (n=28; 28.3%), self-limited epilepsy with centrotemporal spikes (n=19; 19.2%), focal epilepsy with identified lesions (n=17; 17.2%), focal epilepsy of unknown etiology (n=15; 15.2%), epilepsy associated with a genetic syndrome (n=6; 6.1%), epileptic encephalopathy with known etiology (n=5; 5.1%), infantile epileptic spasms syndrome (n=3; 3.0%), epileptic encephalopathy without known cause (n=3; 3.0%), epilepsy with myoclonic atonic seizures syndrome and Lennox-Gastaut syndrome (n=2; 2.0%), and self-limited epilepsy with autonomic seizures (n=1; 1.0%).

The final diagnosis was revised in two (3.1%) patients initially classified as non-epileptic events. Both were subsequently reclassified as epileptic seizures after follow-up. The mean follow-up duration was two years for epileptic patients.

Among patients in Group 2, diagnoses were: vasovagal syncope (n=40; 62.5%), breath-holding spells (n=12; 18.8%), psychogenic non-epileptic seizures (n=3; 4.7%), sleep myoclonus (n=2; 3.1%), night terrors (n=2; 3.1%), tics (n=2; 3.1%), tremor (n=1; 1.6%), and stereotypies (n=2; 3.1%). Among Group 3 patients, causes were hypoglycemia (n=3; 75.0%) and hypocalcemia (n=1; 25.0%).

Patient characteristics at inclusion

Patient characteristics are summarized in Table 1. There were no statistically significant differences in sex distribution between the groups (p=0.108). Age distribution across categories was also not significantly different overall (p=0.295). However, children aged 7-12 years were less likely to present with epileptic seizures compared to the other groups (OR 0.45, 95% CI 0.23-0.87). The mean age was 6.5 years in Group 1 and 6.4 years in Groups 2 and 3.

**Table 1:** Demographic and clinical characteristics of the patients included in the study * P-values calculated using Fisher’s exact test (Freeman-Halton extension). ** Due to small sample size and zero-cell counts † Crude odds ratios comparing Epileptic vs Others, calculated using Haldane-Anscombe correction when zero cell counts were present.

Variable	Epileptic (n=99)	Non-epileptic (n=64)	Occasional (n=4)	p-value*	Crude OR (95% CI)†
Female sex	53 (53.5%)	34 (53.1%)	0	0.108	1.15 (0.62-2.14)
Male sex	46 (46.5%)	30 (46.9%)	4 (100%)	-	0.87 (0.47-1.61)
Age <1 year	10 (10.1%)	4 (6.3%)	0	0.295	1.80 (0.54-5.99)
Age 1-6 years	48 (48.5%)	24 (37.5%)	2 (50%)	-	1.52 (0.81-2.85)
Age 7-12 years	26 (26.3%)	29 (45.3%)	1 (25%)	-	0.45 (0.23-0.87)
Age 13-18 years	15 (15.1%)	7 (10.9%)	1 (25%)	-	1.34 (0.53-3.36)
Neurological family history	46 (46.5%)	2 (3.1%)	0	<0.001	28.64 (6.64-123.47)
Personal neurological history	15 (15.2%)	4 (6.3%)	0	0.167	2.86 (0.90-9.02)
Psychomotor delay	16 (16.2%)	4 (6.3%)	0	0.124	3.08 (0.98-9.67)
Daytime occurrence	74 (74.7%)	62 (96.9%)	4 (100%)	<0.001	0.09 (0.02-0.39)
Nighttime occurrence	25 (25.3%)	2 (3.1%)	0	<0.001	11.15 (2.54-48.88)
Toxic/medication intake	1 (1.0%)	0	0	0.708	2.09 (0.08-51.98)
Stressful/vasovagal situation	2 (2.0%)	47 (73.4%)	0	<0.001	0.01 (0.00-0.04)
Eye rolling	54 (54.5%)	11 (17.2%)	4 (100%)	<0.001	4.24 (2.11-8.51)
Gaze deviation	25 (25.3%)	0	0	<0.001	46.89 (2.80-785.19)
Automatic activity (face/limbs)	23 (23.2%)	4 (6.3%)	0	0.011	4.84 (1.59-14.73)
Vomiting	4 (4.0%)	3 (4.7%)	0	0.896	0.91 (0.20-4.21)
Loss of contact / fixed stare	22 (22.2%)	26 (40.6%)	4 (100%)	<0.001	0.36 (0.18-0.71)
Noisy breathing	13 (13.1%)	0	0	0.008	21.38 (1.25-366.13)
General or partial hypertonia	31 (31.3%)	0	1 (25%)	<0.001	30.54 (4.05-230.19)
Urinary incontinence	4 (4.0%)	0	0	0.245	6.46 (0.34-121.90)
Postictal confusion	67 (67.7%)	0	0	<0.001	284.54 (17.07-4741.56)**
Tongue bite	10 (10.1%)	0	0	0.026	16.07 (0.93-279.11)

Neurological family history was significantly more frequent in the epileptic group (46.5% vs 3.1%, p < 0.001), with a strong association (OR 28.64, 95% CI 6.64-123.47). Personal neurological history and psychomotor developmental delay were more common in Group 1 but did not reach statistical significance (OR 2.86, 95% CI 0.90-9.02; OR 3.08, 95% CI 0.98-9.67, respectively). The patient histories identified in our population included prematurity (n=6), neonatal stroke (n=3), neonatal CNS infection (n=3), and neurosurgical intervention (n=1).

Regarding the circumstances of seizure onset, most events occurred during the daytime across all groups. Daytime occurrence was significantly less associated with epileptic seizures (OR 0.09, 95% CI 0.02-0.39; p < 0.001), whereas nighttime occurrence was strongly associated with epilepsy (OR 11.15, 95% CI 2.54-48.88; p < 0.001). Stressful or vasovagal situations were significantly more frequent in non-epileptic events (OR 0.01, 95% CI 0.00-0.04; p < 0.001).

Semiological features significantly associated with epileptic seizures included eye rolling (OR 4.24, 95% CI 2.11-8.51), gaze deviation (OR 46.89, 95% CI 2.80-785.19), automatic motor activity (OR 4.84, 95% CI 1.59-14.73), noisy breathing (OR 21.38, 95% CI 1.25-366.13), generalized or partial hypertonia (OR 30.54, 95% CI 4.05-230.19), and postictal confusion (OR 284.54, 95% CI 17.07-4741.56), all p < 0.05. Loss of contact or fixed stare was more frequently observed in non-epileptic events (OR 0.36, 95% CI 0.18-0.71; p < 0.001). Urinary incontinence and tongue bite were observed exclusively in epileptic seizures; although statistical testing was performed, the small number of events resulted in wide confidence intervals.

All seizures in Groups 2 and 3 lasted less than 15 minutes, as did the majority of seizures in the epileptic group (92%). Only 8% of patients in Group 1 experienced seizures lasting more than 15 minutes.

Recurrence of seizures during the emergency department stay occurred in 21.2% of patients in Group 1, 14% in Group 2, and 25% in Group 3.

Clinical examination was normal in the majority of patients (84.9% in Group 1, 96.9% in Group 2, and 50% in Group 3). In Group 1, 5% experienced loss of consciousness, 6% had post-seizure deficits, 3% showed hemodynamic disturbances, and 5% had abnormalities on neurological examination. Among patients with occasional seizures, two out of four experienced loss of consciousness, while the remaining patients did not show clinical or hemodynamic abnormalities.

Analysis of the emergency management strategy

Table 2 summarizes the paraclinical examinations prescribed in each group. All patients in Group 1 received a neuropediatric consultation (100%). This consultation occurred within the first 24 hours in 29.3% of cases and during hospitalization in the remaining patients. In contrast, it was requested in 68.8% of Group 2 patients and not at all in Group 3.

**Table 2:** Diagnostic strategy and additional examinations conducted

Variable	Patients having an epileptic seizure (n=99)		Patients having a non-epileptic paroxysmal event (n=64)		Patients having an occasional seizure (n=4)	
	n	%	n	%	n	%
Neuropediatric consultation						
Conducted	99	100	44	68.8	0	0
< 24 hours	29	29.3	32	72.7	0	0
Laboratory tests						
Conducted	52	52.5	18	28.1	4	100
Normal	50	96	18	100	0	0
Abnormal	2	4	0	0	4	100
Capillary glucose	79	79.8	18	28.1	4	100
Toxic						
Conducted	28	28.3	0	0	0	0
Abnormal (alcohol)	10	35.7	0	0	0	0
Lumbar puncture						
Conducted	7	7	0	0	0	0
Normal	7	100	0	0	0	0
Abnormal	0	0	0	0	0	0
Brain CT Scan						
Conducted	10	10.1	1	1.6	0	0
Normal	7	70	1	100	0	0
Abnormal	3	30	0	0	0	0
Brain MRI						
Not conducted	20	20.2	59	92.1	0	0
Emergency < 24h	11	11.1	0	0	0	0
Not urgent	68	68.7	5	50	0	0
Normal	58	73.4	5	100	0	0
Abnormal	21	26.6	0	0	0	0
Fundoscopic examination						
Conducted	2	2	0	0	0	0
Abnormal	0	0	0	0	0	0
ECG						
Conducted	79	79.8	45	70.3	3	75
Abnormal	0	0	1	2.2	1	33.3
EEG						
Conducted	96	96.7	15	23.4	0	0
<24 hours	70	72.9	12	80	0	0
24h - 7 days	23	24	0	0	0	0
>7 days	3	3.1	3	20	0	0
Normal	29	30.2	15	100	0	0
Abnormal	67	69.8	0	0	0	0

Half of the patients in Group 1 (52.5%) underwent comprehensive laboratory testing, including venous glucose, complete electrolyte panel, calcium levels, CRP, and occasionally arterial blood gas, ammonia, liver function, and metabolic panels. Only 4% of these analyses showed abnormalities in Group 1 (increased serum lactate). Additionally, 79.8% of Group 1 patients had capillary glucose testing upon arrival. In Group 2, 28.1% underwent laboratory testing, all of which were normal. In Group 3, all patients underwent laboratory and capillary glucose testing, with abnormalities noted in all cases (three cases of hypoglycemia in diabetic patients and one case of hypocalcemia).

Toxicology screening was performed in 28.3% of Group 1 patients, revealing alcohol-related abnormalities in 35.7% of tested cases. No toxicology screening was conducted in the other Groups.

Lumbar puncture was performed in 7% of patients with epileptic seizures, particularly in cases of prolonged (>15 minutes), repetitive, focal seizures, or suspected central nervous system infection. All lumbar punctures were normal. No lumbar punctures were performed in the other groups.

An ECG was performed in 79.8% of patients in Group 1, 70.3% in Group 2, and 75% in Group 3. One abnormal ECG was recorded in Group 2 (2.2%) and one in Group 3 (33.3%), the latter corresponding to QT prolongation associated with hypocalcemia.

EEG was performed in 96.7% of Group 1 patients. In Group 2, EEG was performed in 23.4% of patients, most frequently within 24 hours (80% of those tested), and all were normal. No EEG was performed in Group 3. In Group 1, 69.8% of EEGs were abnormal.

Cerebral imaging was selectively performed. In Group 1, brain CT was conducted in 10.1% of patients, revealing abnormalities in 30% of cases. Brain MRI was performed in 79.8% of Group 1 patients (11.1% as emergency imaging within 24 hours), with abnormalities identified in 26.6% of cases. Identified lesions included subdural hematoma related to shaken baby syndrome (n=1), brain tumors (n=1), nodular lesions suggestive of neurocysticercosis (n=1), cortical malformations (n=2), arteriovenous malformations (n=2), tumors (n=2), and cysts (n=1). In Group 2, MRI was not performed in 92.1% of patients, and when performed, the results were normal. No cerebral imaging was performed in Group 3.

Other complementary examinations included fundoscopic evaluation, performed in two patients in Group 1 due to suspicion of shaken baby syndrome; none were conducted in the other groups.

## Discussion

History, clinical examination, and differential diagnosis of a first non-febrile convulsive seizure

This study provides a real-world description of children presenting with a first non-febrile convulsive event in a pediatric emergency department, highlighting both diagnostic challenges and variability in management.

The proportion of first epileptic seizures at the pediatric emergency department in Reims from January 2015 to June 2021 was 0.06% of emergency consultations. Our calculated incidence rate was 60.9 per 100,000 person-years, which is consistent with previously reported estimates in Western countries [7-9]. Comparatively, literature from Western countries, such as the study by Benn et al. [7], reported an incidence of newly diagnosed first unprovoked epileptic seizures ranging from 41.1 (95% CI = 35.4-46.8) to 69 per 100,000 person-years [8,9]. However, this estimate should be interpreted with caution, as our study design and inclusion criteria focused on convulsive presentations in an emergency setting rather than population-based incidence.

Age distribution analysis showed a higher prevalence of cases among children aged 1-6 years in the epileptic seizure group, although this did not reach statistical significance. This trend is consistent with previous epidemiological studies showing that seizure incidence is highest in early childhood and decreases with age [7,10].

In our cohort, epileptic seizures accounted for the majority of cases, although non-epileptic paroxysmal events remained frequent. This finding underscores the difficulty of distinguishing epileptic from non-epileptic events in the acute setting and is consistent with previous studies reporting a high proportion of non-epileptic diagnoses among children presenting with suspected seizures [11-13].

Clinical history and seizure semiology were the most informative elements for diagnosis. Features such as eye deviation, eye rolling, generalized or focal hypertonia, and postictal confusion were associated with epileptic seizures, whereas stressful or vasovagal contexts were more frequently observed in non-epileptic events. These findings support the central role of semiology in real-world emergency practice. However, no single clinical or historical feature was sufficient for definitive diagnosis, emphasizing the need for comprehensive clinical assessment [14,15].

Syncope was the most common differential diagnosis and was primarily identified through history-taking. Video recordings, increasingly available through smartphones, may further improve diagnostic accuracy by helping clinicians better characterize paroxysmal events.

Clinical examination alone had limited discriminative value in our cohort, as most patients had a normal examination. Nonetheless, focal neurological deficits or postictal abnormalities, when present, were associated with underlying structural lesions, supporting the importance of careful reassessment after the event. We observed concordance between focal deficits or Todd's paralysis on clinical examination and the diagnosis of intracranial lesions causing focal seizures. This finding aligns with previous studies, such as that of Dayan et al. [16], which explored correlations between history, clinical examination, and diagnosis in first non-provoked seizures across various pediatric emergency departments in the United States from 2005 to 2007.

Recurrence of seizures in the emergency setting was observed in all three groups, highlighting the importance of close monitoring and repeated reassessment. Follow-up also remains essential, as illustrated in our cohort by the reclassification of two initially non-epileptic cases.

Neuropediatrician consultation

In our cohort, neuropediatric consultation was systematically performed in patients with epileptic seizures and in a large proportion of non-epileptic cases, reflecting the complexity of initial diagnostic evaluation in this setting.

This practice is consistent with current recommendations emphasizing that epilepsy diagnosis should be confirmed by a specialist, such as a neurologist, neuropediatrician, or physician trained in epilepsy [3]. Early specialist involvement is essential to ensure accurate diagnosis, appropriate investigations, and timely initiation of management when required. French recommendations further support rapid specialist assessment, particularly in suspected infantile spasms or other epileptic syndromes requiring prompt management [3].

Laboratory testing

Biological tests in this study did not contribute significantly to diagnosis. In Group 1, 52 (52.5%) patients underwent laboratory testing, with 50 (96%) results returning normal.

According to the American Academy of Neurology, the Child Neurology Society, and the American Epilepsy Society, routine laboratory tests are not recommended unless suggested by the clinical history or examination, such as vomiting, diarrhea, or altered consciousness. Toxicological screening should be performed only when specifically indicated. These recommendations highlight the limited diagnostic yield of systematic blood testing in the context of a first seizure [17].

Pediatric studies mainly support targeted testing in infants under six months of age, particularly for hypoglycemia and electrolyte disturbances [17]. Some studies recommend systematic glucose testing [18,19], while others suggest laboratory investigations only when clinical suspicion exists [18,20,21]. 

A study including 107 children presenting with a first non-febrile seizure recommended blood and glucose testing in children with recurrent seizures, gastrointestinal symptoms, age under two years, or altered consciousness [22]. According to the French Health Authority (HAS) guidelines [3], laboratory tests are not required for the positive diagnosis of epilepsy, although capillary glucose, electrolyte levels, and calcium may be prescribed in selected cases.

In our study, laboratory testing was diagnostically useful in only one patient among the 167 included, in whom the clinical context suggested hypocalcemia due to recurrent seizures and previous medical history. However, metabolic causes were identified in the acute symptomatic seizure group, where three patients presented with hypoglycemia and one with hypocalcemia, highlighting the importance of rapid capillary glucose measurement in acute neurological presentations.

Lumbar puncture

Lumbar puncture was rarely performed and did not contribute to diagnosis in our cohort. This is consistent with current recommendations, which restrict its use to cases with suspected central nervous system infection, particularly meningitis or encephalitis, and to young infants in whom clinical signs may be subtle [17].

Brain imaging

Neuroimaging was selectively performed and contributed to diagnosis or management in a substantial proportion of cases, including the identification of serious conditions such as brain tumors requiring urgent neurosurgical intervention. These findings support a targeted approach to imaging and are in keeping with published pediatric data [23,24].

Current French and international recommendations support emergency neuroimaging in cases of focal deficits, prolonged postictal impairment, failure to return to baseline, or suspicion of structural brain lesions [4,17,25,26]. In our practice, emergency CT was often used because of its availability and shorter acquisition time, while MRI was preferred in selected cases according to neurological suspicion, patient age, and feasibility.

Electroencephalogram

EEG was widely used in our cohort and showed a high rate of abnormalities in patients with epileptic seizures, supporting its role in diagnosis, seizure classification, and etiological assessment [17,27,28]. In contrast, the EEG was normal in all cases performed within the non-epileptic group.

Several studies have shown that an EEG performed early after a first seizure has a higher diagnostic yield, especially when obtained within 24 hours [6,29]. Our findings are consistent with these recommendations, as most EEGs were performed within 24 to 72 hours in the epileptic group.

French recommendations also emphasize that EEG findings should always be interpreted in conjunction with the clinical history and specialist assessment [3].

Electrocardiogram

ECG had a limited diagnostic yield in our study, but it remains an important part of the diagnostic workup to exclude cardiac causes such as long QT syndrome or rhythm disorders [3].

Management algorithm

**Figure 2:**
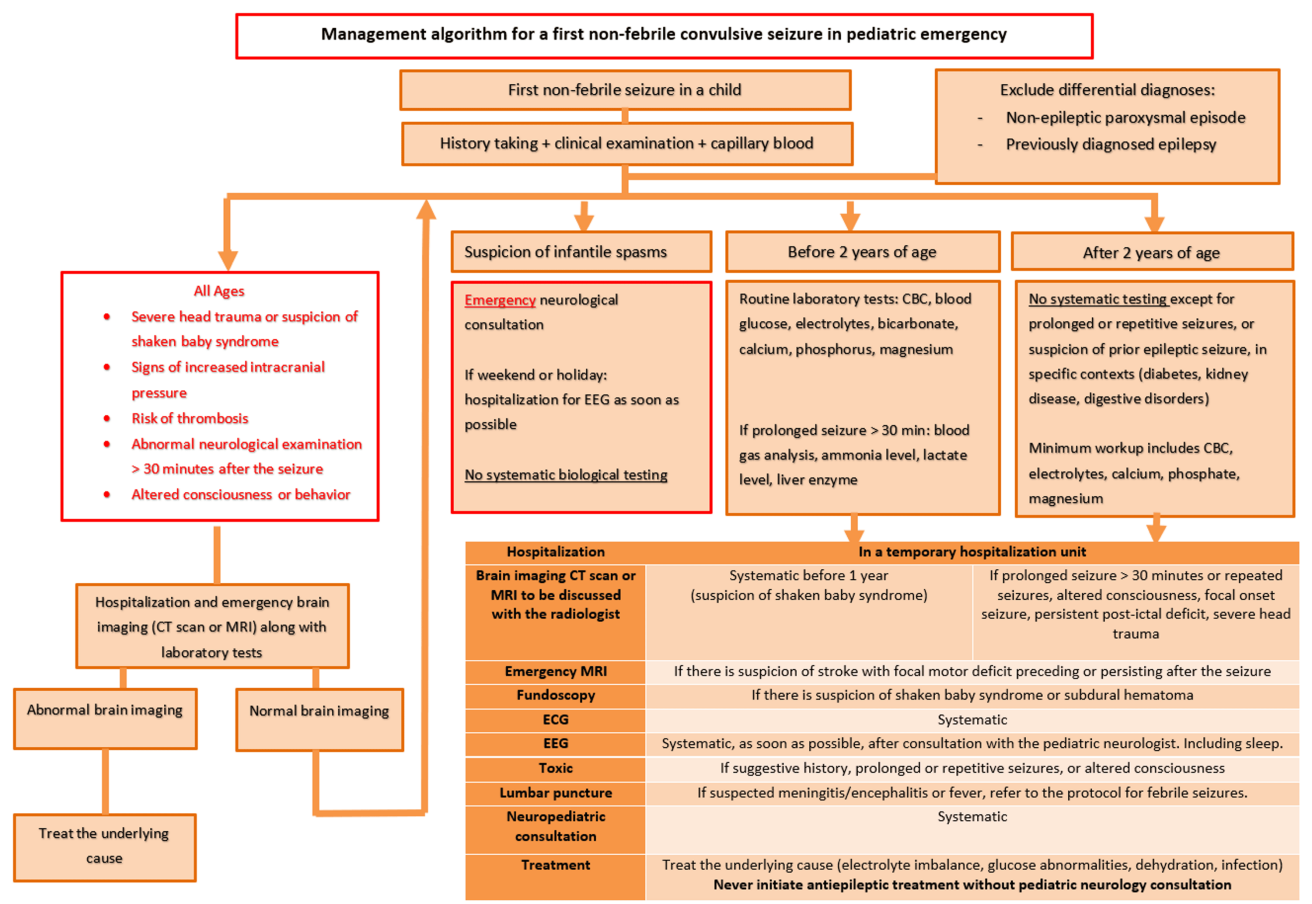
Management algorithm for a first non-febrile convulsive seizure in pediatric emergency This algorithm was developed based on French and international recommendations and existing emergency department protocols [3,4,6,17,26,29].

Considering our findings, French and international recommendations, and existing emergency department protocols from tertiary pediatric centers [3,4,6,17,26,29], our secondary objective was to propose a management algorithm for a first non-febrile convulsive seizure in pediatric emergency settings (Figure 2).

The proposed algorithm does not aim to replace existing guidelines but rather to provide a pragmatic, emergency-oriented synthesis to support clinical decision-making and improve consistency in practice. It translates existing recommendations into a structured step-by-step approach adapted to the constraints of pediatric emergency care.

Discussion of methodology and limitations

Our findings confirm that clinical history and witness description are central to diagnosis, whereas routine laboratory testing has limited diagnostic value. These results are consistent with existing recommendations but also illustrate how these principles are applied in daily practice. However, given the retrospective design and descriptive analysis, these findings should be interpreted cautiously and cannot establish causal relationships.

Among the strengths of our study, we highlight the analysis of real-world practices over a six-year period in a tertiary pediatric emergency department, as well as the availability of follow-up data allowing final diagnostic reclassification in some cases.

Several limitations should be acknowledged. First, the retrospective single-center design may limit generalizability and introduce potential selection bias, particularly as our hospital is a tertiary referral center. Second, reliance on medical records may have led to information bias and diagnostic misclassification, especially in cases where clinical information was incomplete or evolved over time.

Third, the study focused exclusively on convulsive non-febrile events and therefore does not capture the full spectrum of first epileptic seizures in children, particularly non-convulsive presentations such as absence seizures or focal impaired awareness seizures without prominent motor manifestations. In addition, the exclusion of febrile seizures and patients presenting with fever may have reduced the capture of children who were later diagnosed with epilepsy.

The small number of patients in the acute symptomatic seizure group limits the strength of comparisons between groups and precludes firm conclusions regarding this subgroup. Finally, the statistical analysis was primarily descriptive and based on univariate associations, without multivariable modeling. The use of Excel may also limit analytical robustness. Therefore, our findings should be interpreted as exploratory.

This study incorporated French, American, and international guidelines in order to develop an algorithm aligned with current best practices [3,4,6,17,26].

Future studies should evaluate the impact of this algorithm on clinical practice and outcomes. Structured outpatient pathways, including earlier access to EEG and specialist consultation, may also improve care. In addition, strengthening patient and family education after a first seizure could help reduce anxiety and improve understanding of diagnosis, treatment, and safety measures.

## Conclusions

In children presenting with a first non-febrile convulsive event, diagnosis relies primarily on clinical history and seizure semiology, with targeted use of EEG and neuroimaging. In this study, we describe real-world management in a pediatric emergency department, highlighting a broad diagnostic spectrum ranging from benign non-epileptic events to serious neurological conditions requiring urgent intervention. Clinical examination alone was rarely sufficient for diagnosis, underscoring the central role of detailed history-taking and witness descriptions. Several semiological features, including eye deviation, eye rolling, hypertonia, and postictal confusion, were associated with epileptic seizures.

Complementary investigations should be guided by clinical context. Laboratory testing showed limited diagnostic yield overall but remains relevant in selected situations, particularly in infants under two years of age or when metabolic causes are suspected. EEG and neuroimaging were valuable in selected patients for etiological assessment and management decisions.

The proposed management algorithm provides a pragmatic, emergency-oriented framework that may help structure evaluation, reduce unnecessary investigations, and support clinical decision-making. Overall, this study offers practice-based insights into the management of first non-febrile convulsive events and supports a more standardized and targeted approach in pediatric emergency settings.
